# The Report of the Commissioners in Lunacy

**Published:** 1909-08-21

**Authors:** 


### The Hospital

A Journal of

The Medical Sciences and Hospital Administration.

New Series. No. 130, Vol. V. [No. 1202, Vol. XLVI.] SATURDAY, AUGUST 21, 1909.

### The Report of the Commissioners in Lunacy.

The 63rd Report of the Commissioners in Lunacy derives somewhat of unusual interest from its references to the suggestions thrown out last year in the Report of the Royal Commission on the Care and Control of the Feeble-minded. It will be remembered that the Royal Commission, among other recommendations, pronounced for the segregation and restraint of certain classes of persons of defective intellect, especially weak-minded women and girls, with the object of preventing them from becoming parents. It is not surprising to find this recommendation affirmed by the Report, and the wonder is that some measure of this kind has not long ago been enforced. Life is not without its hardships even for those who enter it with a full endowment of mental and bodily integrity, and no great effort of the imagination is required in order to appreciate the extent to which mental weakness enhances the sad side of it. Not least among the mysteries of our existence, from the individual's point of view, is the hard fact that not only the sins, but also the disabilities, of parents are visited so heavily upon their children. But the fact is indubitable, and not to reckon with it is to fail in our duty to posterity. In the ordinary course of nature these inherited sins and disabilities which diminish the efficiency of individuals act as their own corrective according to the laws of survival; but since the humanity of civilised societies does not permit this natural purification of the stock to attain a due fruition, it is reasonable and right that artifice should be invoked to provide a substitute. Certain it is that life without health is not worth living, and if there is one article in which, more than all others, the stewardship of the present generation should be exercised for the benefit of those to come, it is in the provision of good health for the new-born. By comparison with this all benefits conferred upon damaged lives sink into nothing. It is meritorious to provide for shipwrecked mariners, but better far to ordain that they shall not go to sea in crazy vessels.

We recognise that while it is easy to write thus in the abstract, the application to practice of a principle with which no sober person can disagree is fraught with difficulties. For the gradations of mental weakness are so infinitely minute, and its manifestations at times so curiously partial, that it may be impossible to define the limits of normal variation. With children who are manifestly imbecile it is easy to deal. Indeed, the segregation of them is already a condition of their continued existence and involves restraint upon parenthood. But there is a large class of mental defectives who, while not so imbecile as to be unfitted for the simplest and most mechanical labours, yet are morally insane. It is these persons who threaten posterity. It is from their ranks that the majority of girl-mothers of illegitimate children are recruited, for no sense of responsibility or shame is at command to temper the bare animal passions. And what is likely to be the endowment of a child who has such a one for a mother, and for a father one so graceless as to take his mean advantage! For such a girl there is no escape from calamity. Humanly speaking, she is foredoomed to live upon the streets and end her miserable existence in a drunkard's grave, leaving her dismal heritage to others. Certainly something must be done to break this hateful entail of suffering. It may be difficult in the slightest cases to say, "This person is mentally unfit to be a mother"—it is obvious that the segregation of defective males is not nearly so important for the purpose in hand—but, with the increasing attention which is being bestowed upon the medical examination of school children, it ought not to be a matter of insuperable difficulty to weed out these defectives and to place them under such control as is equally necessary for their own welfare and that of the next generation.

The Report affirms also the suggestion of the Royal Commissioners as to the necessity for putting under effective restraint the large number of defectives who commit offences entailing punishment, yet whose mental condition makes punishment an unsuitable method of treatment. Clearly society must protect itself against evil-doers, but the experience of centuries has shown the futility of punishment (in the ordinary sense) as a corrective for the confirmed criminal no less than for the confirmed drunkard. The moral obliquity which produces such offenders finds no antidote in periods of imprisonment. The first moment of freedom is abused for evil purposes, and thus the whole of a life is spent in a gloomy alternation of criminality and gaol. In these cases society is undoubtedly entitled to say, "You have shown that your liberty is a danger to the State; it is therefore forfeit to the State." These people do not require punishment so much as permanent segregation. Not only are they useless citizens and actively dangerous to the welfare of the general public, but in their intervals of enlargement from prison they exert a most pernicious influence upon those in their immediate circle. There must always be a type of the youthful mind particularly susceptible to the allurements of these masters in crime—boys and girls who are attracted in the first instance by the romance and excitement attending criminal exploits, and who find too late how hard it is to retrace their steps. It is not possible, of course, to protect all such people from the consequences of their folly, but something may be done by permanently segregating those confirmed gaol-birds who have proved themselves to be foci of social poisoning. Finally, the Report entirely approves the suggestion of the Royal Commission that the permanent Commission in Lunacy should be strengthened by the appointment of two additional medical members. It points out that modern views of insanity as a disease rather than a mysterious visitation render it appropriate that the personnel of the Lunacy Commission should be predominantly medical. This is an admission of the utmost importance, for it shows that the medical view of insanity is at long last winning its way. As the Report says, insanity is a disease, and the Lunacy Commission, in virtue of its being the central controlling and advisory authority, should be in a position to encourage workers and to assist in determining lines of inquiry designed to elucidate the malady. Such functions can, in the opinion of its present members, only be met by a substantial increase in the proportion of its medical to its legal elements.

